# Results from the Survey of Antibiotic Resistance (SOAR) 2018–21 in Tunisia: data based on CLSI, EUCAST (dose-specific) and pharmacokinetic/pharmacodynamic (PK/PD) breakpoints

**DOI:** 10.1093/jac/dkaf286

**Published:** 2025-11-24

**Authors:** Didem Torumkuney, Leila Slim, Adnene Hammami, Stephen Hawser, Rendani Manenzhe, Anand Manoharan

**Affiliations:** Infectious Diseases Research Unit, GSK, London, UK; Department of Microbiology, Abderrahmen Mami Pneumology Hospital, Ariana, Tunisia; Department of Microbiology, Habib Bourguiba Hospital, University of Sfax, Sfax, Tunisia; Global Affairs, IHMA Europe Sàrl, Rte. De I’Ile-au-Bois 1A, Monthey 1870, Switzerland; Infectious Diseases Research Unit, GSK, Gauteng, South Africa; Infectious Diseases Medical & Scientific Affairs, GSK, Mumbai, India

## Abstract

**Objectives:**

To determine the antibiotic susceptibility of community-acquired respiratory tract infection (CA-RTI) isolates of *Streptococcus pneumoniae* and *Haemophilus influenzae* collected in 2018–21 from two hospitals in Tunisia.

**Methods:**

MICs were determined by CLSI broth microdilution, and susceptibility data were interpreted using CLSI, EUCAST (dose-specific) and pharmacokinetic/pharmacodynamic (PK/PD) breakpoints.

**Results:**

*S. pneumoniae* (*n* = 58) and *H. influenzae* (*n* = 71) isolates were collected; 22.4% of pneumococci were penicillin-susceptible by CLSI oral/EUCAST low-dose breakpoints, but 89.7% were susceptible by EUCAST high-dose/CLSI intravenous administration breakpoints. Susceptibility to ceftriaxone, levofloxacin and moxifloxacin was ≥91.4% by CLSI or PK/PD breakpoints which reduced to 82.8%–87.9% for amoxicillin, amoxicillin/clavulanic acid and cefotaxime. Tetracyclines, macrolides and trimethoprim/sulfamethoxazole were 41.4%–65.5% susceptible, with cefdinir and second-generation cephalosporins less active (24.1%–51.7% susceptible). EUCAST indicated ≥96.6% susceptibility only to high-dose ceftriaxone, moxifloxacin and high-dose levofloxacin. Most *H. influenzae* (66.2%) were β-lactamase negative, of which six and two isolates were ampicillin-resistant following EUCAST and CLSI criteria, respectively. Antibiotic susceptibility was ≥91.5% (CLSI) except for ampicillin (60.6%) and trimethoprim/sulfamethoxazole (77.5%). Susceptibility by EUCAST was lower than CLSI for most other antimicrobials, except for high dose amoxicillin/clavulanic acid (93.0% by EUCAST, 97.2% high dose PK/PD). Both CLSI and EUCAST showed 100% susceptibility to ceftriaxone.

**Conclusions:**

Few therapeutic options with ≥90% susceptibility for the treatment of *S. pneumoniae* from CA-RTIs in Tunisia remain. Although *H. influenzae* isolates displayed higher susceptibility, only ceftriaxone provides 100% coverage for both species following CLSI and EUCAST guidelines. Continued surveillance is important for guiding empiric therapy.

## Introduction

Community-acquired respiratory tract infections (CA-RTIs) are an important world health problem. If treated incorrectly, or in patients with co-morbidities, CA-RTIs can result in hospitalization, with a third of patients with community-acquired pneumonia dying within 12 months of being discharged from hospital.^1^ However, the mortality rate may also have been affected by comorbidities, age and other patient-related risk factors.^1^ Treatment of CA-RTIs relies on empiric antibiotic therapy through the use of national and international guidelines.^2^ A 2018 study of 63 primary care outpatient offices in Tunisia found a high level of inappropriate antibiotic use (75.5%) in the treatment of acute RTIs, as measured by the medication appropriateness index.^3^ The medication appropriateness index assesses various areas of medication prescribing, including the use of an appropriate medication for the disease at the correct dose for the necessary duration, as well as cost.^4,5^ Inappropriate use of antibiotics is linked with high levels of antibiotic resistance.^6^

*S. pneumoniae* and *H. influenzae* are the major bacteria associated with CA-RTIs.^7,8^ Both pathogens have shown increasing resistance to first-line antibiotics such as penicillin and ampicillin.^9,10^ As rates of resistance vary over time and from country to country, up-to-date surveillance data are essential to guide local antibiotic policies.^11^

The Survey of Antibiotic Resistance (SOAR), an international antibiotic resistance surveillance study, focuses on key respiratory pathogens that cause community-acquired infections and has been running since 2002 in the Middle East, Africa, Latin America, Asia-Pacific, Europe and the Commonwealth of Independent States countries.^12^ For this study, recent SOAR data from two hospitals in Tunisia have been analysed to provide an overview of the current state of antibiotic susceptibility of *S. pneumoniae* and *H. influenzae* associated with CA-RTIs.

## Materials and methods

### Ethics

SOAR studies are not human subject studies. During the study, only microorganisms were examined.

### Collaborating centres

Isolates were collected between 2018 and 2021 from two hospitals in Tunisia (Abderrahmen Mami Hospital, Ariana, and Hospital Habib Bourguiba, Sfax).

### Clinical isolates

Isolates of *H. influenzae* and *S. pneumoniae* from CA-RTIs (isolated within 48 h of hospitalization) were sent to a central laboratory (IHMA Europe, Monthey, Switzerland), where they were sub-cultured and re-identified. *H. influenzae* were re-identified by MALDI-TOF MS methodology, and *S. pneumoniae* identity was confirmed by optochin susceptibility and bile solubility. β-lactamase production was determined for each *H. influenzae* isolate by a chromogenic cephalosporin (nitrocefin) disc method. Duplicate isolates from the same patient were not accepted.

### Susceptibility testing

Isolates were evaluated for antibiotic susceptibility using broth microdilution methodology recommended by the CLSI.^13^ Amoxicillin, amoxicillin/clavulanic acid (2:1 ratio as per CLSI guidelines^13,14^), amoxicillin/clavulanic acid (fixed clavulanic acid at 2 mg/L as per the EUCAST guidelines^15^), azithromycin, cefaclor, cefdinir, cefixime, cefotaxime, cefpodoxime, ceftibuten, ceftriaxone, cefuroxime, clarithromycin, levofloxacin, moxifloxacin and trimethoprim/sulfamethoxazole (1:19 ratio) were tested against both respiratory pathogens. In addition, doxycycline, erythromycin and penicillin were tested against *S. pneumoniae* only, and ampicillin was tested against *H. influenzae* only. Susceptibility to the study drugs was calculated based on CLSI, EUCAST (dose-specific) and pharmacokinetic/pharmacodynamic (PK/PD) breakpoints.^14–16^ These breakpoints are given in Tables 1–3. To fully assess antibiotics where high-dose therapies are available, susceptibility using EUCAST criteria was also calculated by combining percentage susceptible and susceptible, increased exposure into the susceptible category as well as dose-dependent PK/PD breakpoints.^15,16^ The antibiotics with high-dose availability assessed in this way were as follows: amoxicillin (0.75–1 g oral, 3× daily), amoxicillin/clavulanic acid (0.875 g amoxicillin/0.125 g clavulanic acid oral, 3× daily), ampicillin [2 g intravenous (IV), 4× daily], penicillin (2.4 g IV, 2 MU 4–6× daily), ceftriaxone (2 g IV, 2× daily), clarithromycin (0.5 g oral, 2× daily), erythromycin (1 g oral or IV, 4× daily), levofloxacin (0.75 g oral 2× daily or 0.4 g IV 3× daily) and trimethoprim/sulfamethoxazole (0.24 g trimethoprim/1.2 g sulfamethoxazole oral or IV, 2× daily).^15^

**Table 1.** CLSI MIC breakpoints (mg/L) used for *S. pneumoniae* and *H. influenzae* isolates

Antimicrobial	*S. pneumoniae*			*H. influenzae*		
	S	I	R	S	I	R
Amoxicillin	≤2	4	≥8	—	—	—
Amoxicillin/clavulanic acid (2:1)^a^	≤2	4	≥8	≤2	4	≥8
Ampicillin	NT	NT	NT	≤1	2	≥4
Azithromycin	≤0.5	1	≥2	≤4	—	—
Cefaclor	≤1	2	≥4	≤8	16	≥32
Cefdinir	≤0.5	1	≥2	≤1	—	—
Cefixime	—	—	—	≤1	—	—
Cefotaxime (non-meningitis)	≤1	2	≥4	≤2	—	—
Cefpodoxime	≤0.5	1	≥2	≤2	—	—
Ceftibuten	—	—	—	≤2	—	—
Ceftriaxone (non-meningitis)	≤1	2	≥4	≤2	—	—
Cefuroxime^b^	≤1	2	≥4	≤4	8	≥16
Clarithromycin	≤0.25	0.5	≥1	≤8	16	≥32
Doxycycline	≤0.25	0.5	≥1	NT	NT	NT
Erythromycin	≤0.25	0.5	≥1	NT	NT	NT
Levofloxacin	≤2	4	≥8	≤2	—	—
Moxifloxacin	≤1	2	≥4	≤1	—	—
Penicillin (2.4 g 2 MU × 4–6 IV)	≤2	4	≥8	NT	NT	NT
Penicillin (oral)	≤0.06	0.12–1	≥2	NT	NT	NT
Tetracycline	≤1	2	≥4	≤2	4	≥8
Trimethoprim/sulfamethoxazole^c^	≤0.5	1–2	≥4	≤0.5	1–2	≥4

—, not applicable; I, intermediate; NT, not tested; R, resistant; S, susceptible.

^a^Amoxicillin/clavulanic acid was tested at a 2:1 amoxicillin to clavulanic acid ratio; breakpoints are expressed as the amoxicillin component.

^b^Breakpoints used are for cefuroxime axetil (oral).

^c^Trimethoprim/sulfamethoxazole was tested at a 1:19 trimethoprim to sulfamethoxazole ratio; breakpoints are expressed as the trimethoprim component.

**Table 2.** EUCAST (dose-specific) MIC breakpoints (mg/L) used for *S. pneumoniae* and *H. influenzae* isolates

Antimicrobial^a^	*S. pneumoniae*		*H. influenzae*	
	S	R	S	R
Amoxicillin (0.5 g × 3 oral)	≤0.5	>1	≤0.001	>2
Amoxicillin (0.75–1 g × 3 oral)	≤1	>1	≤2	>2
Amoxicillin/clavulanic acid (0.5 g/0.125 g × 3 oral)^b^	≤0.5	>1	≤0.001	>2
Amoxicillin/clavulanic acid (0.875 g/0.125 g × 3 oral)^b^	≤1	>1	≤2	>2
Ampicillin (2 g × 3 IV)	NT	NT	≤1	>1
Ampicillin (2 g × 4 IV)	NT	NT	≤1	>1
Azithromycin	≤0.25	>0.5	—	—
Cefaclor	≤0.001	>0.5	—	—
Cefdinir	—	—	—	—
Cefixime	—	—	≤0.12	>0.12
Cefotaxime	≤0.5	>2	≤0.12	>0.12
Cefpodoxime	≤0.25	>0.5	≤0.25	>0.25
Ceftibuten	—	—	≤1	>1
Ceftriaxone (1 g × 1 IV)	≤0.5	>2	≤0.12	>0.12
Ceftriaxone (2 g × 2 IV)	≤2	>2	≤0.12	>0.12
Cefuroxime^c^	≤0.25	>0.5	≤0.001	>1
Clarithromycin (0.25 g × 2 oral)	≤0.25	>0.5		—
Clarithromycin (0.5 g × 2 oral)	≤0.5	>0.5	—	—
Doxycycline	≤1	>2	NT	NT
Erythromycin (0.5 g × 2–4 oral or 0.5 g × 2–4 IV)	≤0.25	>0.5	NT	NT
Erythromycin (1 g × 4 oral or 1 g × 4 IV)	≤0.5	>0.5	NT	NT
Levofloxacin (0.5 g × 2 oral or 0.4 g × 2 IV)	≤0.001	>2	≤0.06	>0.06
Levofloxacin (0.75 g × 2 oral or 0.4 g × 3 IV)	≤2	>2	≤0.06	>0.06
Moxifloxacin	≤0.5	>0.5	≤0.12	>0.12
Penicillin (0.6 g 1 MU × 4 IV)	≤0.06	>2	NT	NT
Penicillin (2.4 g 2 MU × 4–6 IV)	≤2	>2	NT	NT
Tetracycline	≤1	>2	≤2	>2
Trimethoprim/sulfamethoxazole (0.16 g/0.8 g × 2 oral or IV)^d^	≤1	>2	≤0.5	>1
Trimethoprim/sulfamethoxazole (0.24 g/1.2 g × 2 oral or IV)^d^	≤2	>2	≤1	>1

—, not applicable; NT, not tested; R, resistant; S, susceptible.

^a^Where available, susceptibility was assessed using EUCAST higher dosage breakpoints.

^b^Amoxicillin/clavulanic acid was tested at a fixed concentration of 2 mg/L; breakpoints are expressed as the amoxicillin component.

^c^Breakpoints used are for cefuroxime axetil (oral).

^d^Trimethoprim/sulfamethoxazole was tested at a 1:19 trimethoprim to sulfamethoxazole ratio; breakpoints are expressed as the trimethoprim component.

**Table 3.** PK/PD MIC breakpoints (mg/L) used for *S. pneumoniae* and *H. influenzae* isolates

Antimicrobial	*S. pneumoniae* and *H. influenzae*
	S only
Amoxicillin (1.5 g/day)	≤2
Amoxicillin (4 g/day)	≤4
Amoxicillin/clavulanic acid^a^ (1.75 g/0.25 g/day adults; 45 mg/6.4 mg/kg/day children)	≤2
Amoxicillin/clavulanic acid^b^ (4 g/0.25 g/day adults; 90 mg/6.4 mg/kg/day children)	≤4
Ampicillin	—
Penicillin	—
Cefaclor	≤0.5
Cefdinir	≤0.25
Cefditoren	—
Cefixime	≤1
Cefpodoxime	≤0.5
Ceftriaxone	≤1
Cefuroxime^c^	≤1
Azithromycin	≤0.12
Clarithromycin	≤0.25
Erythromycin	≤0.25
Levofloxacin	≤2
Moxifloxacin	≤1
Trimethoprim/sulfamethoxazole^d^	≤0.5

—, not applicable; PK/PD, pharmacokinetic/pharmacodynamic; S, susceptible.

^a^Amoxicillin/clavulanic acid for low dose in adults/children.

^b^Amoxicillin/clavulanic acid for high dose in adults/children.

^c^Breakpoints used are for cefuroxime axetil (oral).

^d^Trimethoprim/sulfamethoxazole was tested at a 1:19 trimethoprim to sulfamethoxazole ratio; breakpoints are expressed as the trimethoprim component.

### Quality control and data analysis

Quality control strains *S. pneumoniae* ATCC 49619, *H. influenzae* ATCC 49247, *H. influenzae* ATCC 49766 and *E. coli* ATCC 32518 were included on each day of testing. Results of susceptibility testing were only accepted if the results of the quality control strains were within the published acceptable range. Differences in susceptibility (using CLSI criteria only) from 2018 to 2021 data reported here compared with Tunisia SOAR data from isolates collected from 2015 to 2018^17^ were assessed for statistical significance with Fisher's exact test using XLSTAT version 2023.1.1.1399. A *P* value of <0.05 was considered statistically significant.

## Results

### *S. pneumoniae* isolates

A total of 58 *S. pneumoniae* isolates were collected from Tunisia between 2018 and 2021. Most isolates came from sputum (*n* = 30, 51.7%), with the remainder from endotracheal aspirate (*n* = 12, 20.7%), middle ear (*n* = 6, 10.3%), blood (*n* = 4, 6.9%), bronchoalveolar lavage (*n* = 3, 5.2%), sinuses (*n* = 1, 1.7%) and unidentified specimens (*n* = 2, 3.4%). Almost half of the isolates (*n* = 26, 44.8%) came from adolescent and adult patients (aged 13–64 years), 23 (39.7%) isolates were from elderly patients (aged ≥65 years) and nine (15.5%) isolates were from paediatric patients (aged ≤12 years).

**Figure 1.**
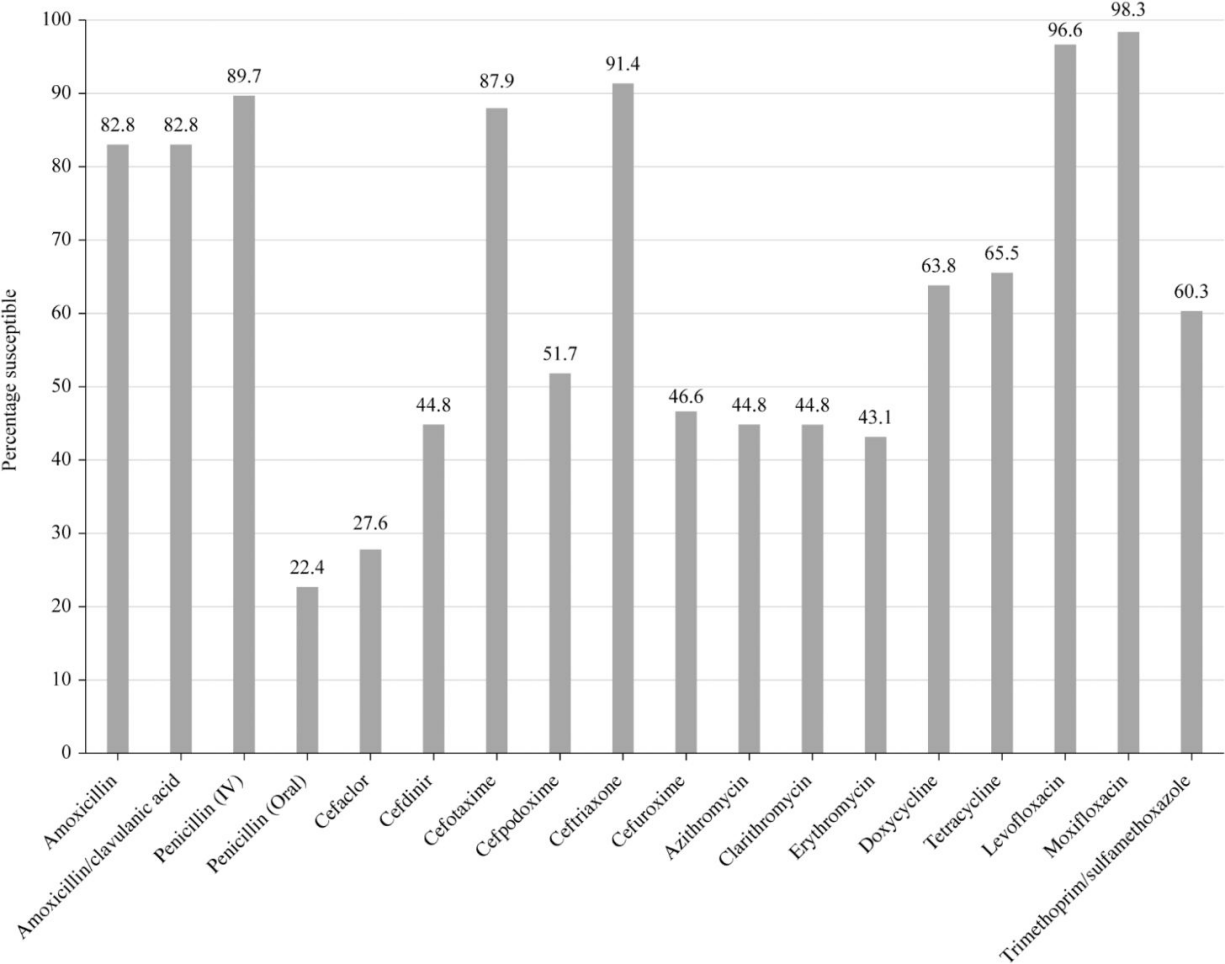
Antibiotic susceptibility rates of *S. pneumoniae* isolates (*n* = 58) from Tunisia based on CLSI breakpoints.

**Figure 2.**
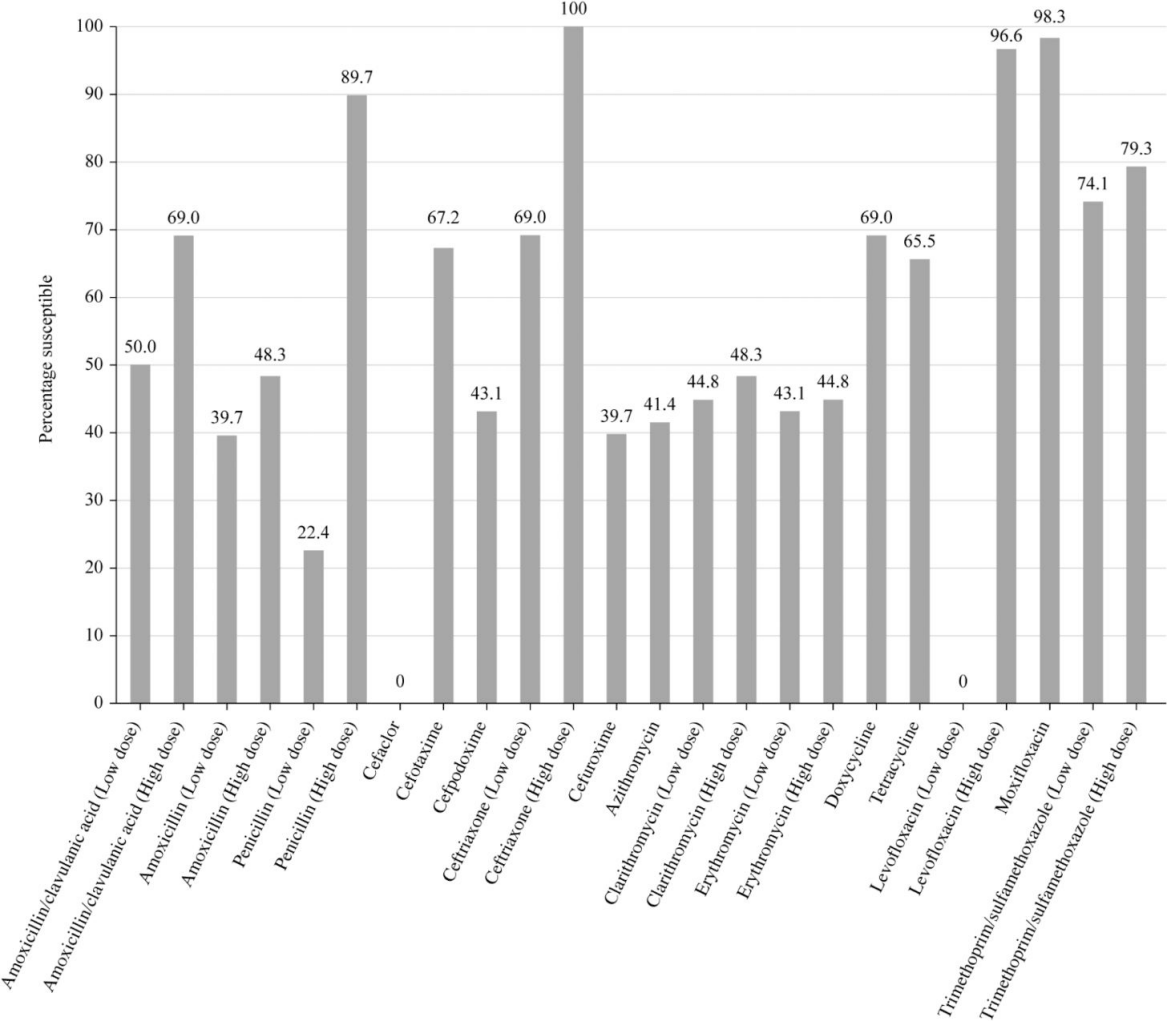
Antibiotic susceptibility rates of *S. pneumoniae* isolates (*n* = 58) from Tunisia based on EUCAST (dose-specific) breakpoints.

Summary MIC, susceptibility and MIC distribution data for all *S. pneumoniae* isolates are given in Tables 4–6 and S1 (available as Supplementary data at *JAC* Online) and shown in Figures 1 and 2.

**Table 4.** MIC and susceptibility data for *S. pneumoniae* isolates (*n* = 58) from Tunisia using CLSI breakpoints

Antimicrobial	MIC (mg/L)			CLSI susceptibility		
	Range	50%	90%	%S	%I	%R
Amoxicillin	≤0.008 to >8	0.5	8	82.8	5.2	12.1
Amoxicillin/clavulanic acid (2:1)	≤0.008 to >8	1	8	82.8	5.2	12.1
Penicillin (2.4 g 2 MU × 4–6 IV)	≤0.008–8	0.5	4	89.7	8.6	1.7
Penicillin (oral)	≤0.008–8	0.5	4	22.4	53.4	24.1
Cefaclor	≤0.015 to >4	>4	>4	27.6	12.1	60.3
Cefdinir	≤0.015 to >8	1	8	44.8	6.9	48.3
Cefixime	≤0.25 to >16	4	>16	—	—	—
Cefotaxime	≤0.008–2	0.25	2	87.9	12.1	0
Cefpodoxime	≤0.015–4	0.5	4	51.7	15.5	32.8
Ceftibuten	≤0.5 to >16	>16	>16	—	—	—
Ceftriaxone	≤0.008–2	0.5	1	91.4	8.6	0
Cefuroxime	≤0.008–8	2	8	46.6	12.1	41.4
Azithromycin	≤0.015 to >16	2	>16	44.8	0	55.2
Clarithromycin	≤0.015 to >16	1	>16	44.8	3.4	51.7
Erythromycin	≤0.015 to >16	2	>16	43.1	1.7	55.2
Doxycycline	0.015 to >4	0.12	>4	63.8	3.4	32.8
Tetracycline	≤0.03 to >4	0.25	>4	65.5	1.7	32.8
Levofloxacin	≤0.12 to >8	1	1	96.6	1.7	1.7
Moxifloxacin	≤0.03–4	0.12	0.12	98.3	0	1.7
Trimethoprim/sulfamethoxazole	≤0.06–8	0.5	4	60.3	19.0	20.7

—, not applicable; I, intermediate; R, resistant; S, susceptible.

**Table 5.** MIC and susceptibility data for *S. pneumoniae* isolates (*n* = 58) from Tunisia using EUCAST (dose-specific) breakpoints

Antimicrobial	MIC (mg/L)			EUCAST susceptibility		
	Range	50%	90%	%S	%I	%R
Amoxicillin (0.5 g × 3 oral)	≤0.008 to >8	0.5	8	50.0	19.0	31.0
Amoxicillin (0.75–1 g × 3 oral)	≤0.008 to >8	0.5	8	69.0	—	31.0
Amoxicillin/clavulanic acid (0.5 g/0.125 g × 3 oral)	≤0.008 to >8	2	>8	39.7	8.6	51.7
Amoxicillin/clavulanic acid (0.875 g/0.125 g × 3 oral)	≤0.008 to >8	2	>8	48.3	—	51.7
Penicillin (0.6 g 1 MU × 4 IV)	≤0.008–8	0.5	4	22.4	67.2	10.3
Penicillin (2.4 g 2 MU × 4–6 IV)	≤0.008–8	0.5	4	89.7	—	10.3
Cefaclor	≤0.015 to >4	>4	>4	0	24.1	75.9
Cefdinir	≤0.015 to >8	1	8	—	—	—
Cefixime	≤0.25 to >16	4	>16	—	—	—
Cefotaxime	≤0.008–2	0.25	2	67.2	32.8	0
Cefpodoxime	≤0.015–4	0.5	4	43.1	8.6	48.3
Ceftibuten	≤0.5 to >16	>16	>16	—	—	—
Ceftriaxone (1 g × 1 IV)	≤0.008–2	0.5	1	69.0	31.0	0
Ceftriaxone (2 g × 2 IV)	≤0.008–2	0.5	1	100	—	0
Cefuroxime	≤0.008–8	2	8	39.7	1.7	58.6
Azithromycin	≤0.015 to >16	2	>16	41.4	3.4	55.2
Clarithromycin (0.25 g × 2 oral)	≤0.015 to >16	1	>16	44.8	3.4	51.7
Clarithromycin (0.5 g × 2 oral)	≤0.015 to >16	1	>16	48.3	—	51.7
Erythromycin (0.5 g × 2–4 oral or 0.5 g × 2–4 IV)	≤0.015 to >16	2	>16	43.1	1.7	55.2
Erythromycin (1 g × 4 oral or 1 g × 4 IV)	≤0.015 to >16	2	>16	44.8	—	55.2
Doxycycline	0.015 to >4	0.12	>4	69.0	6.9	24.1
Tetracycline	≤0.03 to >4	0.25	>4	65.5	1.7	32.8
Levofloxacin (0.5 g × 2 oral or 0.4 g × 2 IV)	≤0.12 to >8	1	1	0	96.6	3.4
Levofloxacin (0.75 g × 2 oral or 0.4 g × 3 IV)	≤0.12 to >8	1	1	96.6	—	3.4
Moxifloxacin	≤0.03–4	0.12	0.12	98.3	—	1.7
Trimethoprim/sulfamethoxazole (0.16 g/0.8 g × 2 oral or IV)	≤0.06–8	0.5	4	74.1	5.2	20.7
Trimethoprim/sulfamethoxazole (0.24 g/1.2 g × 2 oral or IV)	≤0.06–8	0.5	4	79.3	—	20.7

—, not applicable; I, susceptible, increased exposure; R, resistant; S, susceptible.

**Table 6.** Summary MIC and susceptibility data for *S. pneumoniae* (*n* = 58) from Tunisia using PK/PD breakpoints

Antimicrobial	MIC (mg/L)			PK/PD susceptibility
	Range	50%	90%	%S
Amoxicillin (1.5 g/day)	≤0.008 to >8	0.5	8	82.8
Amoxicillin (4 g/day)	≤0.008 to >8	0.5	8	87.9
Amoxicillin/clavulanic acid (1.75 g/0.25 g/day adults; 45 mg/6.4 mg/kg/day children)	≤0.008 to >8	1	8	82.8
Amoxicillin/clavulanic acid (4 g/0.25 g/day adults; 90 mg/6.4 mg/kg/day children)	≤0.008 to >8	1	8	87.9
Penicillin	≤0.008–8	0.5	4	—
Cefaclor	≤0.015 to >4	>4	>4	24.1
Cefdinir	≤0.015 to >8	1	8	41.4
Cefixime	≤0.25 to >16	4	>16	41.4
Cefotaxime	≤0.008–2	0.25	2	—
Cefpodoxime	≤0.015–4	0.5	4	51.7
Ceftibuten	≤0.5 to >16	>16	>16	—
Ceftriaxone	≤0.008–2	0.5	1	91.4
Cefuroxime	≤0.008–8	2	8	46.6
Azithromycin	≤0.015 to >16	2	>16	41.4
Clarithromycin	≤0.015 to >16	1	>16	44.8
Erythromycin	≤0.015 to >16	2	>16	43.1
Doxycycline	0.015 to >4	0.12	>4	63.8
Tetracycline	≤0.03 to >4	0.25	>4	—
Levofloxacin	≤0.12 to >8	1	1	96.6
Moxifloxacin	≤0.03–4	0.12	0.12	98.3
Trimethoprim/sulfamethoxazole	≤0.06–8	0.5	4	60.3

—, not applicable; PK/PD, pharmacokinetic/pharmacodynamic; S, susceptible.

### *S. pneumoniae* susceptibility

The penicillin susceptibility of the pneumococci from Tunisia following CLSI oral or EUCAST low-dose IV breakpoints was 22.4% [53.4% intermediate (CLSI), 24.1% resistant] but susceptibility increased to 89.7% with EUCAST high-dose and CLSI IV breakpoints. Following CLSI breakpoints, the third-generation cephalosporins ceftriaxone and cefotaxime showed similar activity (91.4% and 87.9% susceptible, respectively), but the third-generation cephalosporin cefdinir was less active (44.8% susceptible). Amoxicillin or amoxicillin/clavulanic acid susceptibility was 82.8%, and the second-generation cephalosporins cefpodoxime, cefuroxime and cefaclor showed susceptibility of 51.7%, 46.6% and 27.6%, respectively. In general, susceptibility to the above agents according to PK/PD breakpoints was similar to CLSI except higher dosing for amoxicillin and amoxicillin/clavulanic acid increased susceptibility to 87.9%. The use of EUCAST breakpoints indicated lower susceptibility than with CLSI breakpoints to amoxicillin (69.0% susceptible at high dose), amoxicillin/clavulanic acid (48.3% susceptible at high dose) and all cephalosporins (ranging from 0% susceptible for cefaclor to 67.2% for cefotaxime), except for ceftriaxone (high-dose 100% susceptible; low-dose 69% susceptible). Only high-dose ceftriaxone had >90% susceptibility using EUCAST breakpoints against pneumococci from Tunisia, although high-dose penicillin was close (89.7% susceptible). Activity of between 63.8% and 69.0% was observed with the tetracyclines (doxycycline and tetracycline), and between 41.4% and 48.3%, susceptibility was observed for the macrolides (azithromycin, clarithromycin and erythromycin) by CLSI, PK/PD and EUCAST interpretation. Trimethoprim/sulfamethoxazole susceptibility was 60.3% following CLSI or PK/PD guidelines but 74.1% (low dose) or 79.3% (high dose) by EUCAST interpretation. Moxifloxacin susceptibility was 98.3% following all three breakpoints; similarly, levofloxacin susceptibility was 96.6% by CLSI, PK/PD and EUCAST high-dose breakpoints (Tables 4–6 and S1 and Figures 1 and 2).

### Comparative susceptibility of *S. pneumoniae* collected in 2015–18 and 2018–21

**Figure 3.**
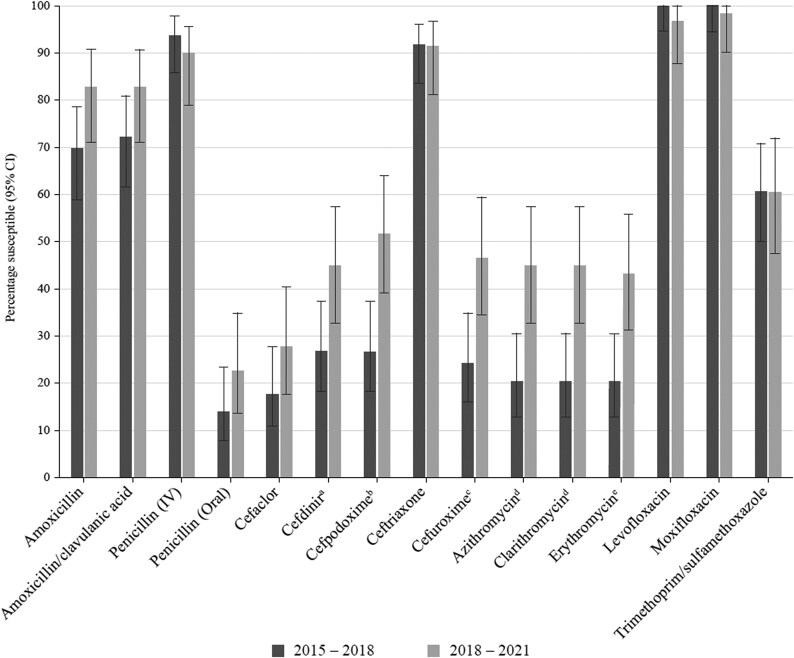
Comparison of antibiotic susceptibility rates of *S. pneumoniae* isolates from Tunisia collected in 2015–18 with isolates collected in 2018–21 (CLSI breakpoints). ^a^Susceptibility was significantly lower in 2015–18 than in 2018–21 (*P* = 0.03). ^b^Susceptibility was significantly lower in 2015–18 than in 2018–21 (*P* = 0.004). ^c^Susceptibility was significantly lower in 2015–18 than in 2018–21 (*P* = 0.01). ^d^Susceptibility was significantly lower in 2015–18 than in 2018–21 (*P* = 0.003). ^e^Susceptibility was significantly lower in 2015–18 than in 2018–21 (*P* = 0.005). CI, confidence interval.

Data from 2015 to 2018 in Tunisia, Kenya and Morocco have previously been published from the SOAR surveillance study^17^ and were compared for mutually tested antibiotics with data from the current study (2018–21) using CLSI breakpoints (Figure 3). There was no significant change in susceptibility to amoxicillin, amoxicillin/clavulanic acid, penicillin, cefaclor, ceftriaxone, fluoroquinolones and trimethoprim/sulfamethoxazole. However, a significant increase in susceptibility to cefdinir (26.6% versus 44.8%, *P* = 0.03), cefpodoxime (26.6% versus 51.7%, *P* = 0.004), cefuroxime (24.1% versus 46.6%, *P* = 0.01) and macrolides [azithromycin: 20.3% versus 44.8%, clarithromycin: 20.3% versus 44.8% (both *P* = 0.003); erythromycin: 20.3% versus 43.1% (*P* = 0.005)] occurred between the two study periods. Although activity of these agents improved, none would be a useful empiric therapeutic option because susceptibility to these agents was between 43.1% and 51.7%.

### *H. influenzae* isolates

A total of 71 *H. influenzae* isolates were collected from Tunisia. Most isolates originated from sputum (*n* = 47, 66.2%). The remaining isolates were from endotracheal aspirate (*n* = 15, 21.1%), bronchoalveolar lavage (*n* = 3, 4.2%), middle ear (*n* = 2, 2.8%) and unidentified specimens (*n* = 4, 5.6%). Many isolates (*n* = 36, 50.7%) came from adolescent and adult patients (aged 13–64 years), 22 (31.0%) isolates were from elderly patients (aged ≥65 years) and 13 (18.3%) isolates were from paediatric patients (aged ≤12 years).

**Figure 4.**
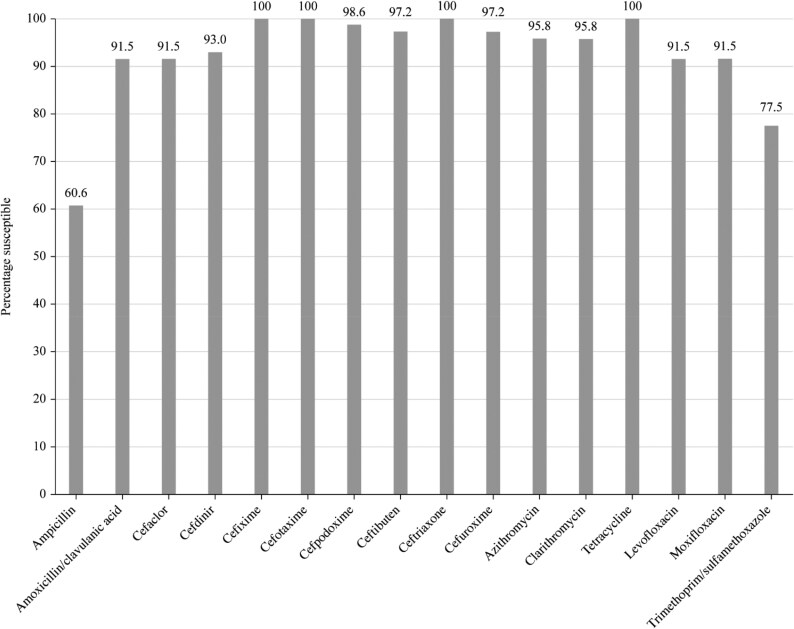
Antibiotic susceptibility rates of *H. influenzae* isolates (*n* = 71) from Tunisia based on CLSI breakpoints.

**Figure 5.**
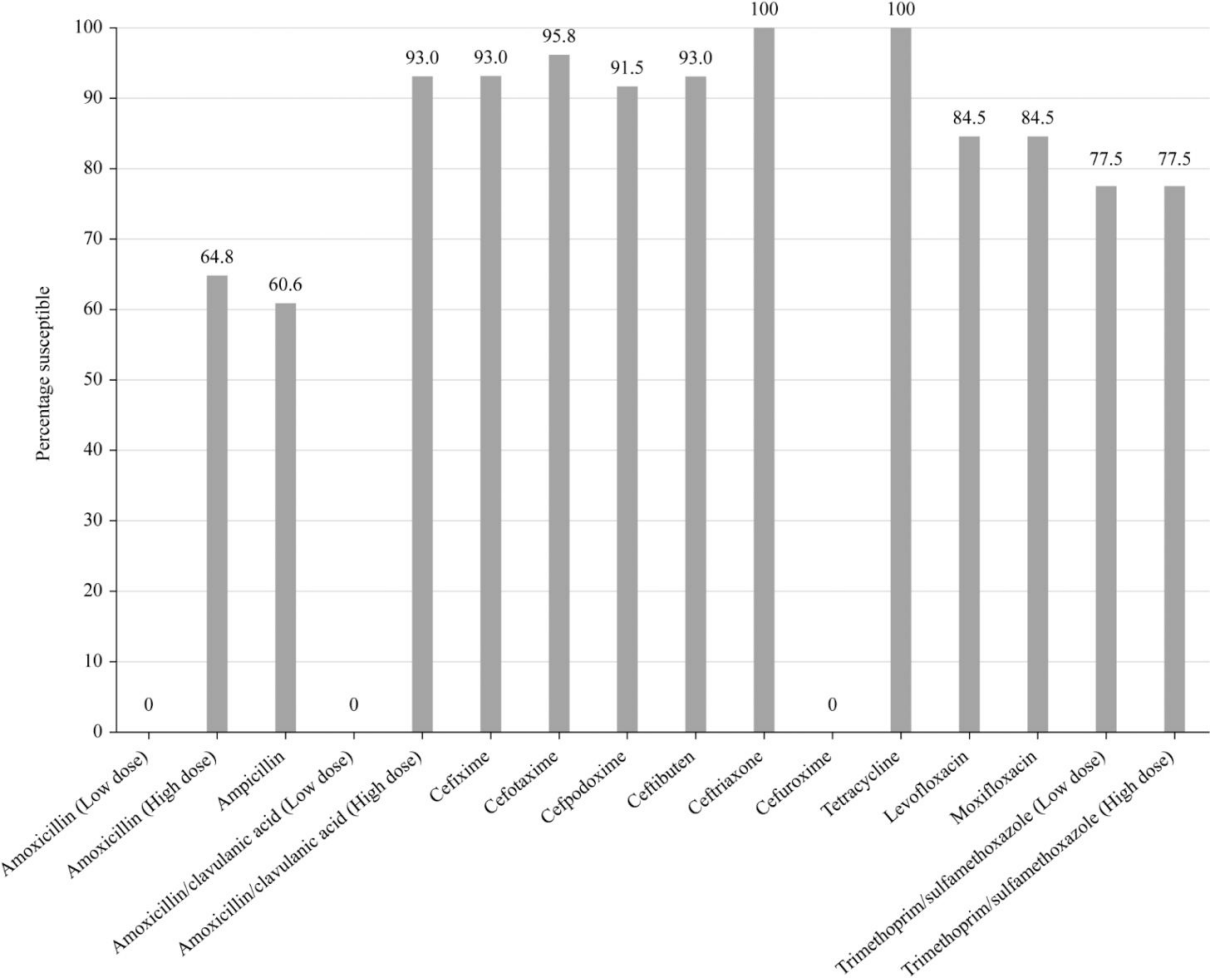
Antibiotic susceptibility rates of *H. influenzae* isolates (*n* = 71) from Tunisia based on EUCAST (dose-specific) breakpoints.

Summary MIC, susceptibility and MIC distribution data for all 71 *H. influenzae* isolates are given in Tables 7–9 and S2 and shown in Figures 4 and 5.

**Table 7.** MIC and susceptibility data for *H. influenzae* isolates (*n* = 71) from Tunisia using CLSI breakpoints

Antimicrobial	MIC (mg/L)			CLSI susceptibility		
	Range	50%	90%	%S	%I	%R
Amoxicillin	≤0.03 to >128	1	64	—	—	—
Ampicillin	≤0.03 to >128	1	128	60.6	5.6	33.8
Amoxicillin/clavulanic acid (2:1)	≤0.03–8	1	2	91.5	5.6	2.8
Cefaclor	≤0.25–32	2	8	91.5	7.0	1.4
Cefdinir	≤0.06–4	0.25	1	93.0	—	—
Cefixime	≤0.008–1	0.03	0.06	100	—	—
Cefotaxime	≤0.002–0.5	0.015	0.06	100	—	—
Cefpodoxime	≤0.015–4	0.06	0.25	98.6	—	—
Ceftibuten	≤0.008 to >4	0.06	0.5	97.2	—	—
Ceftriaxone	≤0.001–0.12	0.004	0.015	100	—	—
Cefuroxime	≤0.03–8	0.5	2	97.2	2.8	0
Azithromycin	≤0.12 to >8	0.5	1	95.8	—	—
Clarithromycin	≤0.25 to >32	4	8	95.8	1.4	2.8
Tetracycline	≤0.12–0.5	0.25	0.5	100	0	0
Levofloxacin	≤0.004 to >8	0.015	0.5	91.5	—	—
Moxifloxacin	≤0.004 to >8	0.015	0.5	91.5	—	—
Trimethoprim/sulfamethoxazole	≤0.008 to >8	0.06	8	77.5	1.4	21.1

—, not applicable; I, intermediate; R, resistant; S, susceptible.

**Table 8.** MIC and susceptibility data for *H. influenzae* isolates (*n* = 71) from Tunisia using EUCAST (dose-specific) breakpoints

Antimicrobial	MIC (mg/L)			EUCAST susceptibility		
	Range	50%	90%	%S	%I	%R
Amoxicillin (0.5 g × 3 oral)	≤0.03 to >128	1	64	0	64.8	35.2
Amoxicillin (0.75–1 g × 3 oral)	≤0.03 to >128	1	64	64.8	—	35.2
Ampicillin	≤0.03 to >128	1	128	60.6	—	39.4
Amoxicillin/clavulanic acid (0.5 g/0.125 g × 3 oral)	≤0.03–8	0.5	2	0	93.0	7.0
Amoxicillin/clavulanic acid (0.875 g/0.125 g × 3 oral)	≤0.03–8	0.5	2	93.0	—	7.0
Cefaclor	≤0.25–32	2	8	—	—	—
Cefdinir	≤0.06–4	0.25	1	—	—	—
Cefixime	≤0.008–1	0.03	0.06	93.0	—	7.0
Cefotaxime	≤0.002–0.5	0.015	0.06	95.8	—	4.2
Cefpodoxime	≤0.015–4	0.06	0.25	91.5	—	8.5
Ceftibuten	≤0.008 to >4	0.06	0.5	93.0	—	7.0
Ceftriaxone	≤0.001–0.12	0.004	0.015	100	—	0
Cefuroxime	≤0.03–8	0.5	2	0	81.7	18.3
Azithromycin	≤0.12 to >8	0.5	1	—	—	—
Clarithromycin	≤0.25 to >32	4	8	—	—	—
Tetracycline	≤0.12–0.5	0.25	0.5	100	—	0
Levofloxacin	≤0.004 to >8	0.015	0.5	84.5	—	15.5
Moxifloxacin	≤0.004 to >8	0.015	0.5	84.5	—	15.5
Trimethoprim/sulfamethoxazole (0.16 g/0.8 g × 2 oral or IV)	≤0.008 to >8	0.06	8	77.5	0	22.5
Trimethoprim/sulfamethoxazole (0.24 g/1.2 g × 2 oral or IV)	≤0.008 to >8	0.06	8	77.5	—	22.5

—, not applicable; I, susceptible, increased exposure; R, resistant; S, susceptible.

**Table 9.** Summary MIC and susceptibility data for *H. influenzae* (*n* = 71) from Tunisia using PK/PD breakpoints

Antimicrobial	MIC (mg/L)			PK/PD susceptibility
	Range	50%	90%	%S
Amoxicillin (1.5 g/day)	≤0.03 to >128	1	64	64.8
Amoxicillin (4 g/day)	≤0.03 to >128	1	64	70.4
Amoxicillin/clavulanic acid (1.75 g/0.25 g/day adults; 45 mg/6.4 mg/kg/day children)	≤0.03 to >128	1	128	91.5
Amoxicillin/clavulanic acid (4 g/0.25 g/day adults; 90 mg/6.4 mg/kg/day children)	≤0.03 to >128	1	128	97.2
Ampicillin	≤0.03–8	1	2	—
Cefaclor	≤0.25–32	2	8	2.8
Cefdinir	≤0.06–4	0.25	1	64.8
Cefixime	≤0.008–1	0.03	0.06	100
Cefotaxime	≤0.002–0.5	0.015	0.06	—
Cefpodoxime	≤0.015–4	0.06	0.25	95.8
Ceftibuten	≤0.008 to >4	0.06	0.5	—
Ceftriaxone	≤0.001–0.12	0.004	0.015	100
Cefuroxime	≤0.03–8	0.5	2	81.7
Azithromycin	≤0.12 to >8	0.5	1	5.6
Clarithromycin	≤0.25 to >32	4	8	2.8
Tetracycline	≤0.12–0.5	0.25	0.5	—
Levofloxacin	≤0.004 to >8	0.015	0.5	91.5
Moxifloxacin	≤0.004 to >8	0.015	0.5	91.5
Trimethoprim/sulfamethoxazole	≤0.008 to >8	0.06	8	77.5

—, not applicable; PK/PD, pharmacokinetic/pharmacodynamic; S, susceptible.

### *H. influenzae* susceptibility

Most isolates of *H. influenzae* were β-lactamase negative but a relatively high percentage (33.8%, 24/71) produced β-lactamases. Six isolates were β-lactamase negative ampicillin-resistant (BLNAR) by EUCAST breakpoints (ampicillin MIC ≥2 mg/L) and two by CLSI breakpoints (ampicillin MIC ≥4 mg/L). Two β-lactamase-positive isolates were found to be ampicillin susceptible. Except for ampicillin (60.6% susceptible) and trimethoprim/sulfamethoxazole (77.5% susceptible), isolates from Tunisia were highly susceptible to all antibiotics tested (≥91.5%) according to CLSI breakpoints. Similar results were obtained when using EUCAST breakpoints, provided high-dose regimens were used for amoxicillin/clavulanic acid. High dosing according to PK/PD breakpoints increased amoxicillin/clavulanic acid susceptibility to 97.2%. Cefuroxime susceptibility of 81.7% was observed using PK/PD breakpoints and 0% was observed using EUCAST breakpoints. Cefdinir susceptibility was 64.8% by PK/PD breakpoints and 93.0% by CLSI, with no breakpoint provision for EUCAST. Fluoroquinolone susceptibility was 84.5% using EUCAST breakpoints compared with 91.5% by both CLSI and PK/PD breakpoints. Cefaclor and macrolide breakpoints are not provided by EUCAST against *H. influenzae*, and in keeping with this, very low cefaclor and macrolide susceptibility (2.8%–5.6%) was observed using PK/PD breakpoints (Tables 7–9 and S2 and Figures 4 and 5).

### Comparative susceptibility of *H. influenzae* collected in 2015–18 and 2018–21

**Figure 6.**
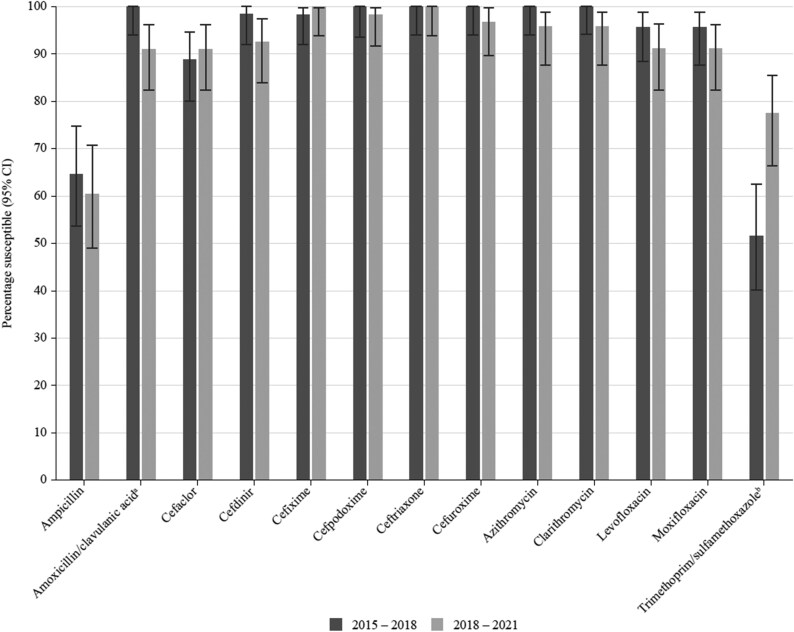
Comparison of antibiotic susceptibility rates of *H. influenzae* isolates from Tunisia collected in 2015–18 with isolates collected in 2018–21 (CLSI breakpoints). ^a^Susceptibility was significantly lower in 2018–21 than in 2015–18 (*P* = 0.012). ^b^Susceptibility was significantly higher in 2018–21 than in 2015–18 (*P* = 0.002).

There was no significant change in susceptibility for most antimicrobials when comparing data from isolates collected in 2015–18^17^ with isolates collected in the current study (2018–21) using CLSI breakpoints (Figure 6). However, susceptibility to amoxicillin/clavulanic acid and azithromycin both decreased between the two study periods (100% versus 91.5% and 100% versus 95.8%, respectively; *P* = 0.012 and *P* = 0.11, respectively). Nevertheless, susceptibility to both agents remained high in 2018–21. Susceptibility to trimethoprim/sulfamethoxazole increased between 2015–18 and 2018–21 (51.4% versus 77.5%, *P* = 0.002), but activity was still relatively low.

## Discussion

SOAR is an ongoing global surveillance study focusing on the two main CA-RTIs pathogens, *S. pneumoniae* and *H. influenzae*, that have been monitored in numerous countries since 2002, including Tunisia, which was involved in the years 2015–18 as well as in the current study. The data presented here are an analysis of the antibiotic susceptibility of *S. pneumoniae* and *H. influenzae* isolates collected from two hospitals in Tunisia between 2018 and 2021. A direct statistical comparison between the two study periods for common antimicrobial agents is also presented.

The penicillin susceptibility results for *S. pneumoniae* in Tunisia 2018–21 clearly show that oral penicillin or low-dose IV penicillin is not an appropriate therapy for CA-RTIs, with only 22.4% of pneumococci susceptible using EUCAST low-dose IV or CLSI oral breakpoints. An even lower level of susceptibility to oral penicillin and low-dose IV penicillin (although not significantly different from 2018 to 2021) was observed between 2015 and 2018 in the previous SOAR study in Tunisia, Kenya and Morocco.^17^ Data from both CLSI and EUCAST guidelines suggest that higher dose IV penicillin could be a better option, though susceptibility was still less than 90% (89.7%).^18^ CLSI breakpoints indicate a slightly lower level of susceptibility to amoxicillin, amoxicillin/clavulanic acid and cefotaxime (82.8%–87.9%), but 91.4% susceptibility to ceftriaxone. Susceptibility to β-lactams using EUCAST breakpoints was 0%–69.0% when following CLSI guidelines, except for high-dose ceftriaxone (100% susceptible). According to PK/PD breakpoints, high-dose amoxicillin or amoxicillin/clavulanic acid increases susceptibility to 87.9%. These contrasting results are most likely based on different criteria or datasets used to determine breakpoints; however, this has important practical implications for clinical laboratories and prescribing decisions. Susceptibility according to either the CLSI or EUCAST guidelines indicated poor activity for macrolides, tetracyclines and trimethoprim/sulfamethoxazole, but excellent activity for fluoroquinolones against *S. pneumoniae* from Tunisia.

Outside of the SOAR study, there are few studies on the antimicrobial susceptibility of CA-RTI isolates from Tunisia. However, a study of invasive (colonizing normally sterile body regions like brain and blood) *S. pneumoniae* from South Tunisia collected between 2012 and 2018 found similar levels of resistance (EUCAST breakpoints) as observed in the current SOAR study to amoxicillin (41.9% versus 31.0%), erythromycin (65.3% versus 55.2%), tetracycline (34.7% versus 32.8%) and trimethoprim/sulfamethoxazole (28.0% versus 20.7%).^19^ In the present study however, only four *S. pneumoniae* were collected from blood and this limits the comparability with the study on invasive pneumococci. Another study investigating pneumococci from immunocompromised patients between 2005 and 2011 confirmed low susceptibility to macrolides (69.5%).^20^

The susceptibility of pneumococci using CLSI breakpoints for isolates previously collected in 2015–18 from Tunisia was compared with susceptibility from the current study (2018–21). As noted above for penicillin, there was no significant difference in susceptibility (CLSI breakpoints) between the two study periods for most antimicrobials. Some cephalosporins (cefdinir, cefpodoxime and cefuroxime) and macrolides showed significantly higher susceptibility in 2018–21 compared with 2015–18, but overall activity was poor for these antimicrobials.

The majority of *H. influenzae* from Tunisia were β-lactamase negative (66.2%), with six being BLNAR according to EUCAST breakpoints and two by CLSI breakpoints. Susceptibility to most antimicrobials was ≥91.5% by CLSI breakpoints, except for ampicillin (60.6% susceptible) and trimethoprim/sulfamethoxazole (77.5% susceptible). Susceptibility with EUCAST breakpoints was lower than CLSI in most cases, with the greatest differences observed with cefuroxime (0% versus 97.2%), cefaclor and macrolides (due to no EUCAST breakpoints being given). Cefaclor and macrolide susceptibility was low according to PK/PD breakpoints. SOAR surveillance from 2015 to 2018 also indicated generally high antibiotic susceptibility in *H. influenzae*, except for ampicillin and trimethoprim/sulfamethoxazole.^17^ There was a statistically significant reduction in the susceptibility of *H. influenzae* to amoxicillin/clavulanic acid and azithromycin between 2015–18 and 2018–21, but susceptibility remained 91.5% and 95.8%, respectively; the reason for this is unclear. In addition, there was a statistically significant increase in susceptibility to trimethoprim/sulfamethoxazole (51.4%–77.5%), but this improved activity would still not be considered of clinical benefit. Cefotaxime resistance in *H. influenzae* from Tunisia was first reported in 2019.^21^ Of the 660 isolates collected between 2013 and 2017, around 1% were cefotaxime-resistant (EUCAST breakpoints).^21^ In the current study, that percentage has risen to 4.2%. Although ceftriaxone susceptibility remained at 100%, it is important to keep a close eye on the development of resistance to third-generation cephalosporins.

Differences in susceptibility between blood and non-blood isolates were evaluated; the corresponding data are not included in this manuscript but were presented at ESCMID Global 2025.^22,23^ For most antibiotics, *S. pneumoniae* and *H. influenzae* showed comparable susceptibility rates between blood and non-blood isolates. However, a subset of non-blood isolates had reduced susceptibility relative to blood isolates, specifically penicillin (oral), trimethoprim/sulfamethoxazole and second-generation cephalosporins (*S. pneumoniae*) and aminopenicillins, trimethoprim/sulfamethoxazole and levofloxacin (*H. influenzae*).

Susceptibility rates may have been influenced by prior antibiotic exposure, introducing a potential limitation in both current and previous SOAR studies. Prior antibiotic treatment could have led to the selection of resistant strains. However, to accurately reflect the real-world resistance landscape experienced by clinicians, patients receiving empirical antibiotic therapy prior to sample collection were not excluded in this study.

In summary, based on these surveillance data, ceftriaxone, fluoroquinolones and high-dose IV penicillin are the most active therapeutic options for the treatment of *S. pneumoniae* originating from CA-RTIs in Tunisia when both EUCAST and CLSI guideline breakpoints are followed. For *H. influenzae*, antimicrobial susceptibility was generally ≥91.5%, except for ampicillin, trimethoprim/sulfamethoxazole and fluoroquinolones. Only ceftriaxone had sufficient activity to cover both CA-RTI pathogens using CLSI and EUCAST breakpoints. Continued surveillance of antibiotic susceptibility in Tunisia is required to regularly assess changes in antimicrobial susceptibility.

## Supplementary Material

dkaf286_Supplementary_Data

## Supplementary data

Tables S1 and S2 are available as Supplementary data at *JAC* Online.
