# Supplementary material for: Results from the Survey of Antibiotic Resistance (SOAR) 2018–21 in Tunisia: data based on CLSI, EUCAST (dose-specific) and pharmacokinetic/pharmacodynamic (PK/PD) breakpoints
Source: J Antimicrob Chemother. 2025 Nov 24;80(Suppl 3):iii53–67. doi: 10.1093/jac/dkaf286 (PMC12641129; doi:10.1093/jac/dkaf286)
Supplement: dkaf286_Supplementary_Data [file dkaf286_supplementary_data.docx]

**Results from the Survey of Antibiotic Resistance (SOAR) 2018 – 21 in Tunisia: data based on CLSI, EUCAST (dose-specific) and pharmacokinetic/pharmacodynamic (PK/PD) breakpoints**

**Authors:** Didem TORUMKUNEY^1^, Leila SLIM^2^, Adnene HAMMAMI^3^, Stephen HAWSER^4^, Rendani MANENZHE^5†^, Anand MANOHARAN^6*^

**Affiliations:** ^1^GSK, London, UK; ^2^Department of Microbiology, Abderrahmen Mami Pneumology Hospital, Ariana, Tunisia; ^3^Department of Microbiology, Habib Bourguiba Hospital, University of Sfax, Tunisia; ^4^IHMA Europe Sàrl, Rte. De I’Ile-au-Bois 1A, 1870 Monthey, Switzerland; ^5^GSK, Gauteng, South Africa; ^6^Infectious Diseases Medical & Scientific Affairs, GSK, Mumbai, India.

^†^Affiliation at the time of the study

*Corresponding author. E-mail: anand.x.manoharan@gsk.com

**Running title:** Survey of Antibiotic Resistance (SOAR) in Tunisia in 2018 – 21

**Supplementary Table 1.** MIC distribution data for *S. pneumoniae* isolates (*n* = 58) from Tunisia

|  |  | Number of isolates at MIC (mg/L) | | | | | | | | | | | | | | | | | | | | |
| --- | --- | --- | --- | --- | --- | --- | --- | --- | --- | --- | --- | --- | --- | --- | --- | --- | --- | --- | --- | --- | --- | --- |
| Antimicrobial |  | ≤0.008 | ≤0.015 | 0.015 | ≤0.03 | 0.03 | ≤0.06 | 0.06 | ≤0.12 | 0.12 | ≤0.25 | 0.25 | ≤0.5 | 0.5 | 1 | 2 | 4 | >4 | 8 | >8 | 16 | >16 |
| AMX | N | 5 | – | 7 | – | 1 | – | – | – | 5 | – | 6 | – | 5 | 11 | 8 | 3 | – | 6 | 1 | – | – |
|  | Cum. % | 8.6 | – | 20.7 | – | 22.4 | – | – | – | 31.0 | – | 41.4 | – | 50.0 | 69.0 | 82.8 | 87.9 | – | 98.3 | 100 | – | – |
|  | % | 8.6 | – | 12.1 | – | 1.7 | – | – | – | 8.6 | – | 10.3 | – | 8.6 | 19.0 | 13.8 | 5.2 | – | 10.3 | 1.7 | – | – |
| AMC (2:1) | N | 7 | – | 5 | – | 1 | – | – | – | 6 | – | 5 | – | 2 | 14 | 8 | 3 | – | 6 | 1 | – | – |
|  | Cum. % | 12.1 | – | 20.7 | – | 22.4 | – | – | – | 32.8 | – | 41.4 | – | 44.8 | 69.0 | 82.8 | 87.9 | – | 98.3 | 100 | – | – |
|  | % | 12.1 | – | 8.6 | – | 1.7 | – | – | – | 10.3 | – | 8.6 | – | 3.4 | 24.1 | 13.8 | 5.2 | – | 10.3 | 1.7 | – | – |
| AMC [2 mg/L] | N | 7 | – | – | – | 6 | – | – | – | 1 | – | 5 | – | 4 | 5 | 6 | 7 | – | 11 | 6 | – | – |
|  | Cum. % | 12.1 | – | – | – | 22.4 | – | – | – | 24.1 | – | 32.8 | – | 39.7 | 48.3 | 58.6 | 70.7 | – | 89.7 | 100 | – | – |
|  | % | 12.1 | – | – | – | 10.3 | – | – | – | 1.7 | – | 8.6 | – | 6.9 | 8.6 | 10.3 | 12.1 | – | 19.0 | 10.3 | – | – |
| AZM | N | – | 3 | – | – | 8 | – | 13 | – | – | – | – | – | 2 | – | 5 | 2 | – | – | – | – | 25 |
|  | Cum. % | – | 5.2 | – | – | 19.0 | – | 41.4 | – | – | – | – | – | 44.8 | – | 53.4 | 56.9 | – | – | – | – | 100 |
|  | % | – | 5.2 | – | – | 13.8 | – | 22.4 | – | – | – | – | – | 3.4 | – | 8.6 | 3.4 | – | – | – | – | 43.1 |
| CEC | N | – | 1 | – | – | – | – | – | – | 2 | – | – | – | 11 | 2 | 7 | 2 | 33 | – | – | – | – |
|  | Cum. % | – | 1.7 | – | – | – | – | – | – | 5.2 | – | – | – | 24.1 | 27.6 | 39.7 | 43.1 | 100 | – | – | – | – |
|  | % | – | 1.7 | – | – | – | – | – | – | 3.4 | – | – | – | 19.0 | 3.4 | 12.1 | 3.4 | 56.9 | – | – | – | – |
| CDR | N | – | 2 | – | – | 5 | – | 7 | – | 3 | – | 7 | – | 2 | 4 | 5 | 10 | – | 12 | 1 | – | – |
|  | Cum. % | – | 3.4 | – | – | 12.1 | – | 24.1 | – | 29.3 | – | 41.4 | – | 44.8 | 51.7 | 60.3 | 77.6 | – | 98.3 | 100 | – | – |
|  | % | – | 3.4 | – | – | 8.6 | – | 12.1 | – | 5.2 | – | 12.1 | – | 3.4 | 6.9 | 8.6 | 17.2 | – | 20.7 | 1.7 | – | – |
| CFM | N | – | – | – | – | – | – | – | – | – | 17 | – | – | 6 | 1 | 4 | 4 | – | 6 | – | 11 | 9 |
|  | Cum. % | – | – | – | – | – | – | – | – | – | 29.3 | – | – | 39.7 | 41.4 | 48.3 | 55.2 | – | 65.5 | – | 84.5 | 100 |
|  | % | – | – | – | – | – | – | – | – | – | 29.3 | – | – | 10.3 | 1.7 | 6.9 | 6.9 | – | 10.3 | – | 19.0 | 15.5 |
| CTX | N | 7 | – | 6 | – | 4 | – | 6 | – | 1 | – | 6 | – | 9 | 12 | 7 | – | – | – | – | – | – |
|  | Cum. % | 12.1 | – | 22.4 | – | 29.3 | – | 39.7 | – | 41.4 | – | 51.7 | – | 67.2 | 87.9 | 100 | – | – | – | – | – | – |
|  | % | 12.1 | – | 10.3 | – | 6.9 | – | 10.3 | – | 1.7 | – | 10.3 | – | 15.5 | 20.7 | 12.1 | – | – | – | – | – | – |
| CPD | N | – | 7 | – | – | 6 | – | 2 | – | 7 | – | 3 | – | 5 | 9 | 9 | 10 | – | – | – | – | – |
|  | Cum. % | – | 12.1 | – | – | 22.4 | – | 25.9 | – | 37.9 | – | 43.1 | – | 51.7 | 67.2 | 82.8 | 100 | – | – | – | – | – |
|  | % | – | 12.1 | – | – | 10.3 | – | 3.4 | – | 12.1 | – | 5.2 | – | 8.6 | 15.5 | 15.5 | 17.2 | – | – | – | – | – |
| CTB | N | – | – | – | – | – | – | – | – | – | – | – | 1 | – | – | 6 | 8 | – | 7 | – | 2 | 34 |
|  | Cum. % | – | – | – | – | – | – | – | – | – | – | – | 1.7 | – | – | 12.1 | 25.9 | – | 37.9 | – | 41.4 | 100 |
|  | % | – | – | – | – | – | – | – | – | – | – | – | 1.7 | – | – | 10.3 | 13.8 | – | 12.1 | – | 3.4 | 58.6 |
| CRO | N | 5 | – | 6 | – | 4 | – | 5 | – | 2 | – | 5 | – | 13 | 13 | 5 | – | – | – | – | – | – |
|  | Cum. % | 8.6 | – | 19 | – | 25.9 | – | 34.5 | – | 37.9 | – | 46.6 | – | 69.0 | 91.4 | 100 | – | – | – | – | – | – |
|  | % | 8.6 | – | 10.3 | – | 6.9 | – | 8.6 | – | 3.4 | – | 8.6 | – | 22.4 | 22.4 | 8.6 | – | – | – | – | – | – |
| CXM | N | 5 | – | 5 | – | 4 | – | 1 | – | 2 | – | 6 | – | 1 | 3 | 7 | 14 | – | 10 | – | – | – |
|  | Cum. % | 8.6 | – | 17.2 | – | 24.1 | – | 25.9 | – | 29.3 | – | 39.7 | – | 41.4 | 46.6 | 58.6 | 82.8 | – | 100 | – | – | – |
|  | % | 8.6 | – | 8.6 | – | 6.9 | – | 1.7 | – | 3.4 | – | 10.3 | – | 1.7 | 5.2 | 12.1 | 24.1 | – | 17.2 | – | – | – |
| CLR | N | – | 17 | – | – | 7 | – | – | – | 1 | – | 1 | – | 2 | 5 | 3 | – | – | 1 | – | – | 21 |
|  | Cum. % | – | 29.3 | – | – | 41.4 | – | – | – | 43.1 | – | 44.8 | – | 48.3 | 56.9 | 62.1 | – | – | 63.8 | – | – | 100 |
|  | % | – | 29.3 | – | – | 12.1 | – | – | – | 1.7 | – | 1.7 | – | 3.4 | 8.6 | 5.2 | – | – | 1.7 | – | – | 36.2 |
| DOX | N | – | – | 1 | – | 9 | – | 16 | – | 11 | – | – | – | 2 | 1 | 4 | 8 | 6 | – | – | – | – |
|  | Cum. % | – | – | 1.7 | – | 17.2 | – | 44.8 | – | 63.8 | – | – | – | 67.2 | 69.0 | 75.9 | 89.7 | 100 | – | – | – | – |
|  | % | – | – | 1.7 | – | 15.5 | – | 27.6 | – | 19.0 | – | – | – | 3.4 | 1.7 | 6.9 | 13.8 | 10.3 | – | – | – | – |
| ERY | N | – | 8 | – | – | 14 | – | 2 | – | – | – | 1 | – | 1 | 2 | 4 | 2 | – | 2 | – | – | 22 |
|  | Cum. % | – | 13.8 | – | – | 37.9 | – | 41.4 | – | – | – | 43.1 | – | 44.8 | 48.3 | 55.2 | 58.6 | – | 62.1 | – | – | 100 |
|  | % | – | 13.8 | – | – | 24.1 | – | 3.4 | – | – | – | 1.7 | – | 1.7 | 3.4 | 6.9 | 3.4 | – | 3.4 | – | – | 37.9 |
| LVX | N | – | – | – | – | – | – | – | 2 | – | – | – | – | 17 | 35 | 2 | 1 | – | – | 1 | – | – |
|  | Cum. % | – | – | – | – | – | – | – | 3.4 | – | – | – | – | 32.8 | 93.1 | 96.6 | 98.3 | – | – | 100 | – | – |
|  | % | – | – | – | – | – | – | – | 3.4 | – | – | – | – | 29.3 | 60.3 | 3.4 | 1.7 | – | – | 1.7 | – | – |
| MXF | N | – | – | – | 2 | – | – | 23 | – | 29 | – | 1 | – | 2 | – | – | 1 | – | – | – | – | – |
|  | Cum. % | – | – | – | 3.4 | – | – | 43.1 | – | 93.1 | – | 94.8 | – | 98.3 | – | – | 100 | – | – | – | – | – |
|  | % | – | – | – | 3.4 | – | – | 39.7 | – | 50.0 | – | 1.7 | – | 3.4 | – | – | 1.7 | – | – | – | – | – |
| PEN | N | 6 | – | 7 | – | – | – | – | – | 8 | – | 5 | – | 3 | 15 | 8 | 5 | – | 1 | – | – | – |
|  | Cum. % | 10.3 | – | 22.4 | – | – | – | – | – | 36.2 | – | 44.8 | – | 50.0 | 75.9 | 89.7 | 98.3 | – | 100 | – | – | – |
|  | % | 10.3 | – | 12.1 | – | – | – | – | – | 13.8 | – | 8.6 | – | 5.2 | 25.9 | 13.8 | 8.6 | – | 1.7 | – | – | – |
| TET | N | – | – | – | 1 | – | – | 7 | – | 17 | – | 11 | – | 1 | 1 | 1 | 1 | 18 | – | – | – | – |
|  | Cum. % | – | – | – | 1.7 | – | – | 13.8 | – | 43.1 | – | 62.1 | – | 63.8 | 65.5 | 67.2 | 69 | 100 | – | – | – | – |
|  | % | – | – | – | 1.7 | – | – | 12.1 | – | 29.3 | – | 19.0 | – | 1.7 | 1.7 | 1.7 | 1.7 | 31.0 | – | – | – | – |
| SXT | N | – | – | – | – | – | 6 | – | – | 5 | – | 17 | – | 7 | 8 | 3 | 7 | – | 5 | – | – | – |
|  | Cum. % | – | – | – | – | – | 10.3 | – | – | 19.0 | – | 48.3 | – | 60.3 | 74.1 | 79.3 | 91.4 | – | 100 | – | – | – |
|  | % | – | – | – | – | – | 10.3 | – | – | 8.6 | – | 29.3 | – | 12.1 | 13.8 | 5.2 | 12.1 | – | 8.6 | – | – | – |

–, not applicable; AMC, amoxicillin/clavulanic acid; AMX, amoxicillin; AZM, azithromycin; CDR, cefdinir; CEC, cefaclor; CFM, cefixime; CLR, clarithromycin; CPD, cefpodoxime; CRO, ceftriaxone; CTB, ceftibuten; CTX, cefotaxime; Cum, cumulative; CXM, cefuroxime; DOX, doxycycline; ERY, erythromycin; LVX, levofloxacin; MIC, minimum inhibitory concentration; MXF, moxifloxacin; PEN, penicillin; SXT, trimethoprim/sulfamethoxazole; TET, tetracycline.

Bold vertical bars in table correspond to the CLSI-susceptible breakpoints.

**Supplementary Table 2.** MIC distribution data for *H. influenzae* isolates (*n* = 71) from Tunisia

|  |  | Number of isolates at MIC (mg/L) | | | | | | | | | | | | | | | | | | | | | | | | | | | | | | |
| --- | --- | --- | --- | --- | --- | --- | --- | --- | --- | --- | --- | --- | --- | --- | --- | --- | --- | --- | --- | --- | --- | --- | --- | --- | --- | --- | --- | --- | --- | --- | --- | --- |
| Antimicrobial |  | ≤0.001 | ≤0.002 | 0.002 | ≤0.004 | 0.004 | ≤0.008 | 0.008 | ≤0.015 | 0.015 | ≤0.03 | 0.03 | ≤0.06 | 0.06 | ≤0.12 | 0.12 | ≤0.25 | 0.25 | 0.5 | 1 | 2 | 4 | >4 | 8 | >8 | 16 | 32 | >32 | 64 | 128 | >128 |  |
| AMX | N | – | – | – | – | – | – | – | – | – | 3 | – | – | – | – | – | – | 9 | 21 | 8 | 5 | 4 | – | 2 | – | 1 | 7 | – | 4 | 4 | 3 |  |
|  | Cum. % | – | – | – | – | – | – | – | – | – | 4.2 | – | – | – | – | – | – | 16.9 | 46.5 | 57.7 | 64.8 | 70.4 | – | 73.2 | – | 74.6 | 84.5 | – | 90.1 | 95.8 | 100 |  |
|  | % | – | – | – | – | – | – | – | – | – | 4.2 | – | – | – | – | – | – | 12.7 | 29.6 | 11.3 | 7.0 | 5.6 | – | 2.8 | – | 1.4 | 9.9 | – | 5.6 | 5.6 | 4.2 |  |
| AMC (2:1) | N | – | – | – | – | – | – | – | – | – | 2 | – | – | – | – | – | – | 5 | 24 | 20 | 14 | 4 | – | 2 | – | – | – | – | – | – | – |  |
|  | Cum. % | – | – | – | – | – | – | – | – | – | 2.8 | – | – | – | – | – | – | 9.9 | 43.7 | 71.8 | 91.5 | 97.2 | – | 100 | – | – | – | – | – | – | – |  |
|  | % | – | – | – | – | – | – | – | – | – | 2.8 | – | – | – | – | – | – | 7.0 | 33.8 | 28.2 | 19.7 | 5.6 | – | 2.8 | – | – | – | – | – | – | – |  |
| AMC [2mg/L] | N | – | – | – | – | – | – | – | – | – | 1 | – | – | 1 | – | 3 | – | 17 | 26 | 11 | 7 | 4 | – | 1 | – | – | – | – | – | – | – |  |
|  | Cum. % | – | – | – | – | – | – | – | – | – | 1.4 | – | – | 2.8 | – | 7.0 | – | 31.0 | 67.6 | 83.1 | 93.0 | 98.6 | – | 100 | – | – | – | – | – | – | – |  |
|  | % | – | – | – | – | – | – | – | – | – | 1.4 | – | – | 1.4 | – | 4.2 | – | 23.9 | 36.6 | 15.5 | 9.9 | 5.6 | – | 1.4 | – | – | – | – | – | – | – |  |
| AMP | N | – | – | – | – | – | – | – | – | – | 3 | – | – | – | – | 7 | – | 20 | 5 | 8 | 4 | 2 | – | 3 | – | 2 | 5 | – | 3 | 5 | 4 |  |
|  | Cum. % | – | – | – | – | – | – | – | – | – | 4.2 | – | – | – | – | 14.1 | – | 42.3 | 49.3 | 60.6 | 66.2 | 69.0 | – | 73.2 | – | 76.1 | 83.1 | – | 87.3 | 94.4 | 100 |  |
|  | % | – | – | – | – | – | – | – | – | – | 4.2 | – | – | – | – | 9.9 | – | 28.2 | 7.0 | 11.3 | 5.6 | 2.8 | – | 4.2 | – | 2.8 | 7.0 | – | 4.2 | 7.0 | 5.6 |  |
| AZM | N | – | – | – | – | – | – | – | – | – | – | – | – | – | 4 | – | – | 5 | 27 | 29 | 3 | – | – | 1 | 2 | – | – | – | – | – | – |  |
|  | Cum. % | – | – | – | – | – | – | – | – | – | – | – | – | – | 5.6 | – | – | 12.7 | 50.7 | 91.5 | 95.8 | – | – | 97.2 | 100 | – | – | – | – | – | – |  |
|  | % | – | – | – | – | – | – | – | – | – | – | – | – | – | 5.6 | – | – | 7.0 | 38.0 | 40.8 | 4.2 | – | – | 1.4 | 2.8 | – | – | – | – | – | – |  |
| CEC | N | – | – | – | – | – | – | – | – | – | – | – | – | – | – | – | 2 | – | – | 10 | 26 | 18 | – | 9 | – | 5 | 1 | – | – | – | – |  |
|  | Cum. % | – | – | – | – | – | – | – | – | – | – | – | – | – | – | – | 2.8 | – | – | 16.9 | 53.5 | 78.9 | – | 91.5 | – | 98.6 | 100 | – | – | – | – |  |
|  | % | – | – | – | – | – | – | – | – | – | – | – | – | – | – | – | 2.8 | – | – | 14.1 | 36.6 | 25.4 | – | 12.7 | – | 7.0 | 1.4 | – | – | – | – |  |
| CDR | N | – | – | – | – | – | – | – | – | – | – | – | 5 | – | – | 8 | – | 33 | 13 | 7 | 4 | 1 | – | – | – | – | – | – | – | – | – |  |
|  | Cum. % | – | – | – | – | – | – | – | – | – | – | – | 7.0 | – | – | 18.3 | – | 64.8 | 83.1 | 93.0 | 98.6 | 100 | – | – | – | – | – | – | – | – | – |  |
|  | % | – | – | – | – | – | – | – | – | – | – | – | 7.0 | – | – | 11.3 | – | 46.5 | 18.3 | 9.9 | 5.6 | 1.4 | – | – | – | – | – | – | – | – | – |  |
| CFM | N | – | – | – | – | – | 4 | – | – | 10 | – | 40 | – | 10 | – | 2 | – | 1 | 2 | 2 | – | – | – | – | – | – | – | – | – | – | – |  |
|  | Cum. % | – | – | – | – | – | 5.6 | – | – | 19.7 | – | 76.1 | – | 90.1 | – | 93.0 | – | 94.4 | 97.2 | 100 | – | – | – | – | – | – | – | – | – | – | – |  |
|  | % | – | – | – | – | – | 5.6 | – | – | 14.1 | – | 56.3 | – | 14.1 | – | 2.8 | – | 1.4 | 2.8 | 2.8 | – | – | – | – | – | – | – | – | – | – | – |  |
| CTX | N | – | 13 | – | – | 12 | – | 6 | – | 20 | – | 11 | – | 5 | – | 1 | – | 1 | 2 | – | – | – | – | – | – | – | – | – | – | – | – |  |
|  | Cum. % | – | 18.3 | – | – | 35.2 | – | 43.7 | – | 71.8 | – | 87.3 | – | 94.4 | – | 95.8 | – | 97.2 | 100 | – | – | – | – | – | – | – | – | – | – | – | – |  |
|  | % | – | 18.3 | – | – | 16.9 | – | 8.5 | – | 28.2 | – | 15.5 | – | 7 | – | 1.4 | – | 1.4 | 2.8 | – | – | – | – | – | – | – | – | – | – | – | – |  |
| CPD | N | – | – | – | – | – | – | – | 3 | – | – | 18 | – | 28 | – | 11 | – | 5 | 3 | 2 | – | 1 | – | – | – | – | – | – | – | – | – |  |
|  | Cum. % | – | – | – | – | – | – | – | 4.2 | – | – | 29.6 | – | 69.0 | – | 84.5 | – | 91.5 | 95.8 | 98.6 | – | 100 | – | – | – | – | – | – | – | – | – |  |
|  | % | – | – | – | – | – | – | – | 4.2 | – | – | 25.4 | – | 39.4 | – | 15.5 | – | 7.0 | 4.2 | 2.8 | – | 1.4 | – | – | – | – | – | – | – | – | – |  |
| CTB | N | – | – | – | – | – | 3 | – | – | – | – | 12 | – | 33 | – | 7 | – | 7 | 4 | – | 3 | 1 | 1 | – | – | – | – | – | – | – | – |  |
|  | Cum. % | – | – | – | – | – | 4.2 | – | – | – | – | 21.1 | – | 67.6 | – | 77.5 | – | 87.3 | 93 | – | 97.2 | 98.6 | 100 | – | – | – | – | – | – | – | – |  |
|  | % | – | – | – | – | – | 4.2 | – | – | – | – | 16.9 | – | 46.5 | – | 9.9 | – | 9.9 | 5.6 | – | 4.2 | 1.4 | 1.4 | – | – | – | – | – | – | – | – |  |
| CRO | N | 6 | – | 10 | – | 26 | – | 17 | – | 7 | – | 1 | – | 2 | – | 2 | – | – | – | – | – | – | – | – | – | – | – | – | – | – | – |  |
|  | Cum. % | 8.5 | – | 22.5 | – | 59.2 | – | 83.1 | – | 93.0 | – | 94.4 | – | 97.2 | – | 100 | – | – | – | – | – | – | – | – | – | – | – | – | – | – | – |  |
|  | % | 8.5 | – | 14.1 | – | 36.6 | – | 23.9 | – | 9.9 | – | 1.4 | – | 2.8 | – | 2.8 | – | – | – | – | – | – | – | – | – | – | – | – | – | – | – |  |
| CXM | N | – | – | – | – | – | – | – | – | – | 3 | – | – | – | – | – | – | 9 | 34 | 12 | 7 | 4 | – | 2 | – | – | – | – | – | – | – |  |
|  | Cum. % | – | – | – | – | – | – | – | – | – | 4.2 | – | – | – | – | – | – | 16.9 | 64.8 | 81.7 | 91.5 | 97.2 | – | 100 | – | – | – | – | – | – | – |  |
|  | % | – | – | – | – | – | – | – | – | – | 4.2 | – | – | – | – | – | – | 12.7 | 47.9 | 16.9 | 9.9 | 5.6 | – | 2.8 | – | – | – | – | – | – | – |  |
| CLR | N | – | – | – | – | – | – | – | – | – | – | – | – | – | – | – | 2 | – | – | 2 | 3 | 40 | – | 21 | – | 1 | – | 2 | – | – | – |  |
|  | Cum. % | – | – | – | – | – | – | – | – | – | – | – | – | – | – | – | 2.8 | – | – | 5.6 | 9.9 | 66.2 | – | 95.8 | – | 97.2 | – | 100 | – | – | – |  |
|  | % | – | – | – | – | – | – | – | – | – | – | – | – | – | – | – | 2.8 | – | – | 2.8 | 4.2 | 56.3 | – | 29.6 | – | 1.4 | – | 2.8 | – | – | – |  |
| LVX | N | – | – | – | 3 | – | – | 3 | – | 50 | – | 4 | – | – | – | – | – | 1 | 3 | 1 | – | – | – | – | 6 | – | – | – | – | – | – |  |
|  | Cum. % | – | – | – | 4.2 | – | – | 8.5 | – | 78.9 | – | 84.5 | – | – | – | – | – | 85.9 | 90.1 | 91.5 | – | – | – | – | 100 | – | – | – | – | – | – |  |
|  | % | – | – | – | 4.2 | – | – | 4.2 | – | 70.4 | – | 5.6 | – | – | – | – | – | 1.4 | 4.2 | 1.4 | – | – | – | – | 8.5 | – | – | – | – | – | – |  |
| MXF | N | – | – | – | 3 | – | – | 12 | – | 38 | – | 6 | – | 1 | – | – | – | 1 | 3 | 1 | – | – | – | 4 | 2 | – | – | – | – | – | – |  |
|  | Cum. % | – | – | – | 4.2 | – | – | 21.1 | – | 74.6 | – | 83.1 | – | 84.5 | – | – | – | 85.9 | 90.1 | 91.5 | – | – | – | 97.2 | 100 | – | – | – | – | – | – |  |
|  | % | – | – | – | 4.2 | – | – | 16.9 | – | 53.5 | – | 8.5 | – | 1.4 | – | – | – | 1.4 | 4.2 | 1.4 | – | – | – | 5.6 | 2.8 | – | – | – | – | – | – |  |
| TET | N | – | – | – | – | – | – | – | – | – | – | – | – | – | 4 | – | – | 55 | 12 | – | – | – | – | – | – | – | – | – | – | – | – |  |
|  | Cum. % | – | – | – | – | – | – | – | – | – | – | – | – | – | 5.6 | – | – | 83.1 | 100 | – | – | – | – | – | – | – | – | – | – | – | – |  |
|  | % | – | – | – | – | – | – | – | – | – | – | – | – | – | 5.6 | – | – | 77.5 | 16.9 | – | – | – | – | – | – | – | – | – | – | – | – |  |
| SXT | N | – | – | – | – | – | 3 | – | – | 2 | – | 8 | – | 26 | – | 10 | – | 6 | – | – | 1 | 3 | – | 6 | 6 | – | – | – | – | – | – |  |
|  | Cum. % | – | – | – | – | – | 4.2 | – | – | 7.0 | – | 18.3 | – | 54.9 | – | 69.0 | – | 77.5 | – | – | 78.9 | 83.1 | – | 91.5 | 100 | – | – | – | – | – | – |  |
|  | % | – | – | – | – | – | 4.2 | – | – | 2.8 | – | 11.3 | – | 36.6 | – | 14.1 | – | 8.5 | – | – | 1.4 | 4.2 | – | 8.5 | 8.5 | – | – | – | – | – | – |  |

–, not applicable; AMC, amoxicillin/clavulanic acid; AMX, amoxicillin; Antim., antimicrobial; AZM, azithromycin; CDR, cefdinir; CEC, cefaclor; CFM, cefixime; CLR, clarithromycin; CPD, cefpodoxime; CRO, ceftriaxone; CTB, ceftibuten; CTX, cefotaxime; CXM, cefuroxime; LVX, levofloxacin; MXF, moxifloxacin; SXT, trimethoprim/sulfamethoxazole; TET, tetracycline.

Bold vertical bars in table correspond to the CLSI-susceptible breakpoints.
